# The Buffering Effect of Machiavellianism on the Relationship Between Role Conflict and Counterproductive Work Behavior

**DOI:** 10.3389/fpsyg.2018.01776

**Published:** 2018-09-21

**Authors:** Jun Zhao, Sufang Xiao, Jianghua Mao, Wenxing Liu

**Affiliations:** ^1^School of Public Administration, Zhongnan University of Economics and Law, Wuhan, China; ^2^School of Business Administration, Zhongnan University of Economics and Law, Wuhan, China

**Keywords:** role conflict, counterproductive work behavior, emotional exhaustion, Machiavellianism, job stressor

## Abstract

Considering the destructive effects of counterproductive work behavior (CWB) in the workplace, scholars have put much effort into revealing its antecedents. The purpose of this paper is to examine how Machiavellianism helps mitigate the effect of role conflict on CWB in China. Using data collected from three phases, this research revealed that role conflict had a positive effect on CWB *via* emotional exhaustion. Machiavellianism moderated the relationship between role conflict and emotional exhaustion, such that this relationship got weaker for employees with higher Machiavellianism. Furthermore, Machiavellianism moderated the relationship between role conflict and CWB *via* emotional exhaustion, as such, it became weaker for employees with high Machiavellianism.

## Introduction

Counterproductive work behavior (CWB) is a form of intentional behavior that can harm an organization or its members (Spector and Fox, 2002). It includes verbal abuse, lying, theft, and sabotage (Greenberg, 1990; Ambrose et al., 2002; Spector et al., 2006). The pervasiveness and high cost of CWB have been reported to harm businesses all over the world (Penney et al., 2011; Chiu et al., 2015). To mitigate the negative effects of CWB, much effort has been put into revealing its antecedents across both environmental and individual domains (Colbert et al., 2004; Mount et al., 2006; Penney et al., 2011). For example, CWB is believed to occur in response to strong work stressors, stressful work environment, and the consequent negative emotions (Spector and Fox, 2005; Bolton et al., 2012). Personality traits, such as emotional stability, agreeableness, and conscientiousness, also have a convincing relation with CWB (Berry et al., 2007; Penney et al., 2011). However, despite their efforts, many scholars (Colbert et al., 2004; Sprung and Jex, 2012; Meurs et al., 2013) have cautioned that our understanding of the antecedents of CWB is still far from perfect due to a variety of reasons.

First, we still lack enough knowledge about the joint effect of work conditions and personality traits on CWB (Colbert et al., 2004). Although certain recent studies have stressed the moderating role of personality traits on the relationship between job stressors and employee CWB (e.g., Colbert et al., 2004; Sprung and Jex, 2012; Meurs et al., 2013; Zhou et al., 2014), they focused mainly on a part of the “big five” personality traits, such as conscientiousness. Few studies have paid attention to exploring the role of other typical personality traits (e.g., the dark triad personality traits, Paulhus and Williams, 2002), which are equally essential in understanding the antecedents of CWB (Spain et al., 2014; DeShong et al., 2015). Second, questions remain regarding whether the results of CWB could be generalized to non-Western countries (e.g., China). Several scholars have noted that employee CWB may have varying predictors across different cultures (Rotundo and Xie, 2008; Peng, 2012), indicating that a non-Western study is needed to reach a more comprehensive understanding of the antecedences of CWB.

The present study was designed to address these gaps based on the theory of conservation of resources (CORs, Hobfoll, 1989), which is one of the leading theoretical models of stress domains (Sun and Chen, 2017). We use COR theory to examine how job stressor (i.e., role conflict) predicts employee CWB. Role conflict consists of the incongruities and incompatibilities of the requirements of a role that impinges upon role performance (Rizzo et al., 1970). We focus on role conflict because it has been regarded as one of the common job stressors (Spector and Jex, 1998; Perrewé et al., 2004) and has been found to be linked with several dysfunctional outcomes (Schaubroeck et al., 1989; Perrewé et al., 2004). Drawing on COR theory, we argue that the complex requirements caused by role conflict consume employees’ resources, leading to emotional exhaustion among employees and eventually triggers CWB among them (e.g., wasting time or withholding effort) to conserve their limited resources (Hobfoll et al., 2003).

Second, we propose that Machiavellianism plays a buffering role in role conflict–CWB relationships because it can utilize effective coping strategies toward preventing resource loss (Penney et al., 2011). Machiavellianism refers to individuals who fixate on self-interest and ignore the correctness of the means they use to achieve their aims (Dahling et al., 2009). Since Machiavellian individuals ignore moral norms and approve behaviors that harm others for their own benefit (Dahling et al., 2009), previous studies have pointed the positive link between Machiavellianism and CWB (Giacalone and Knouse, 1990; O’Boyle et al., 2012). Since employees with high Machiavellianism show better environmental adaptability and may be more able to cope with resource losses caused by role conflict (Christie and Geis, 1970; Penney et al., 2011), we suggest that such employees will be more likely to experience less emotional exhaustion and consequently engage less in CWB from the perspective of COR.

We attempt to explore whether and how role conflict affects CWB in a non-Western culture (i.e., China) by echoing the prior calls about revealing the role conflict–CWB association in different cultures (e.g., Rotundo and Xie, 2008; Peng, 2012). We target on China because China is believed to have several significant cultural differences such as power distance (Liang and Gong, 2013) and traditionality (Farh et al., 2007) with Western cultures. It will be possible that because employees in China will value organizational expectations and are more inclined to obey and fulfil supervisors’ expectations unconditionally (Farh et al., 2007), they may be more likely to have a higher tolerance for role conflict. Therefore, we believe that examining whether and how role conflict affects CWB in China is meaningful.

By so doing, the present research contributes to CWB and Machiavellianism literature in multiple ways. First, our examination of whether role conflict has a positive effect on CWB *via* emotional exhaustion, contributes to CWB literature by providing evidence of the relationship between job stressors, emotional exhaustion, and CWB in a non-Western setting (i.e., China). Second, by exploring the buffering role of Machiavellianism on the relationship between role conflict and employee CWB *via* emotional exhaustion, we provide an integrated conception about the joint effect of environmental and individual antecedents on CWB. Finally, although Machiavellianism has been linked with CWB, our research provides the new insight that Machiavellianism could better cope with role conflict, thus enhancing our understanding of Machiavellianism at the workplace.

## Theory and Hypotheses

### Conservation of Resources Theory and Counterproductive Work Behaviors

Conservation of resource theory emphasizes the important role of resources in preventing psychological stress or strained outcomes in individuals (Halbesleben and Buckley, 2004; Van Woerkom et al., 2016). Hobfoll (1989) proposed that these resources could be “objects, personal characteristics, conditions, or energies that are valued by the individual” (p. 516) or anything that helps an individual attain their goals (Halbesleben et al., 2014). Employees can gain resources both from organizations (e.g., social and/or financial support) and individual differences (e.g., self-efficacy). The psychological resources that employees have secured can help individuals achieve their goals better (Kammeyer-Mueller et al., 2016). According to COR theory, resource loss leads to psychological strain (Lee and Ashforth, 1996), such as emotional exhaustion, so resource investing is a basic method for individuals to gain resources (Ng and Feldman, 2012).

Conservation of resource theory serves as a fundamental explanation of how work conditions and even personal factors affect employee CWB (Bolton et al., 2012; Chiu et al., 2015). For example, based on COR theory, Chiu et al. (2015) argued that role stressors would render employees to pay more attention to their stressful work conditions, resulting in information overload, energy depletion, and subsequent CWB. However, although they attempted to explore the relationship between work conditions (i.e., role stressors) and CWB, the underlying mechanism has remained under explored. As a supplement, Bolton et al. (2012) proposed that employees engaged in CWB protect their resources to cope with the resource loss caused by emotional exhaustion. Furthermore, scholars have also used COR theory to explain how individual factors affect CWB (Penney et al., 2011). For example, Penney et al. (2011) argued that individual differences served as personal resources that helped employees meet work demands and reach their goals. They found that emotional stability was significantly related to CWB. However, the relationship between conscientiousness and CWB was not significant, indicating a complex relationship between personality and CWB. To advance our understanding about the antecedents of CWB, the present research explores how work conditions (i.e., role conflict), personality traits (i.e., Machiavellianism), and their interaction influence employee CWB.

### Role Conflict, Emotional Exhaustion, and Counterproductive Work Behavior

Role conflict is a central component of job stressor, which refers to the incongruities and incompatibilities of the requirements of a role that impinge upon role performance (Rizzo et al., 1970). Individuals constantly monitor and evaluate the events that occur in their workplace (Lazarus, 1991) and consider events that threaten their well-being as job stressors. This subsequently causes physiological and behavioral changes and motivates employees to take a series of actions that may be detrimental to the organization or its members. CWB is one of the behavioral responses to job stress (Spector and Fox, 2005). Researches have provided much evidence on the positive relationship between role conflict and CWB. For example, Chen and Spector (1992) found that role conflict was significantly correlated with forms of CWB such as sabotage, interpersonal aggression, hostility, complaints, theft, and intention to quit. Hauge et al. (2009) identified role conflict as an effective situational factor predicting workplace bullying.

In explaining how role conflict affects CWB, the emotional exhaustion perspective has been regarded as an important explanation. Consistent with such previous studies, we propose that role conflict has a positive effect on employee CWB, as it may provoke employees’ emotional exhaustion. Emotional exhaustion is “a psychological strain that is a response to chronic work stressors” (Halbesleben and Buckley, 2004; Sun and Chen, 2017, p. 1474). It describes the “feelings of being emotionally overextended and exhausted by one’s work” (Maslach and Jackson, 1981, p. 101). Following COR theory, we propose that role conflict threatens and diminishes employees’ resources, which leads to emotional exhaustion. First, role conflict causes employees to focus their attention on different or conflicting expectations (Faucett et al., 2013), which causes an information overload or expectation confusion, resulting in loss of emotional resources. Second, the conflicting expectations caused by role conflict needs employees to consume much more resources (e.g., time and energy) to deal with, thus resulting in emotional exhaustion. In addition, role conflict can cause a decline in sense of control and autonomy because of too many assignments from different supervisors. Researches have come up with considerable evidence that role conflict is associated with the loss of emotional resources, such as environmental frustration (Keenan and Newton, 1984) and tension or anxiety (Jackson and Schuler, 1985). Thus, when employees are faced with high level of role conflict, they become depressed and exhausted from dealing with different requirements and expectations.

Conservation of resource theory posits that individuals strive to retain and protect the resources they value (Hobfoll et al., 2003). When role conflict causes a loss of valued resources, such as emotional and energy resources, sense of control and autonomy, employees often adopt defensive strategies to prevent further losing of resources. CWB is one set of coping behaviors to protect and restore resources, which may relieve employees’ negative psychological states (Krischer et al., 2010; Reynolds et al., 2015). On the one hand, employees can reduce resource investment (e.g., of time and energy) by reducing the effort or shrinking the responsibility to protect and maintain psychological resources. On the other hand, engaging in CWB helps employees release the psychological strain caused by role conflict and symbolically restore a sense of control (Bennett, 1998). It costs emotional resources for employees to cope with role conflict (Bolton et al., 2012). COR theory assumes that people put efforts into preventing further resource losses by saving their remaining resources through physical or psychological withdrawal from the stressful situation (Ito and Brotheridge, 2003) or other people (Leiter, 1993). Thus, in order to prevent further resource losses, employees will be more likely to engage in CWB to protect their existing resources. Therefore, we propose:

*Hypothesis 1*: *Role conflict has a positive effect on employee CWB via emotional exhaustion.*

### Buffering Effect of Machiavellianism

In recent years, studies about dark and socially aversive personality traits have gradually increased, so many have paid attention to Machiavellianism (Paulhus and Williams, 2002; Lee and Ashton, 2005). Machiavellianism is commonly defined as an individual’s behavioral tendency to take advantage of others to attain personal goals (Christie and Geis, 1970; Wilson et al., 1996; Linton and Wiener, 2001). Calhoon (1969) considered a Machiavellian administrator to be one who uses aggressive, manipulative, exploitative, and devious methods to achieve personal and organizational objectives. Such methods are primarily undertaken according to the perceived feasibility while giving less consideration to the feelings, needs, and/or rights of others.

A conventional viewpoint is that the Machiavellian employee is the “bad apple” in an organization (Tang et al., 2008; Kish-Gephart et al., 2010; Jonason et al., 2014). Individuals with high Machiavellian tendencies pay more attention to personal interest, exhibit opportunistic behaviors to maximize their benefits, and engage in unethical behaviors. Mudrack (1993) examined 10 forms of workplace behaviors of dubious ethical nature and found that such behaviors were internally consistent and clearly correlated with Machiavellianism. Tang et al. (2008) found that the Machiavellianism personality trait was significantly correlated with negative behaviors, such as resource abuse, theft, corruption, and deception.

Although several studies have illustrated the negative effects of Machiavellianism at the workplace, our understanding of Machiavellianism is still far from enough, beyond the ethical category (Tang et al., 2008). In the organizational context, the Machiavellian employees show a certain degree of complexity. On the one hand, employees with high Machiavellianism are perceived as bad apples and are even suspected to be troublemakers. While facing urgent decisions, they often ignore others’ feelings, interests, and needs, so they are labeled as being “cold blooded” (Deluga, 2001). However, on the other hand, several scholars also argued that Machiavellianism only describes individual’s specific behavior methods and strategies; thus, it cannot be designated as good or bad by definition (Paulhus and Williams, 2002; Lee and Ashton, 2005).

We propose that Machiavellianism moderates the relationship between role conflict and emotional exhaustion. Specifically, Machiavellianism can buffer the positive effect of role conflict on emotional exhaustion. According to COR theory, role conflict indicates the complicated requirements and expectations of employees that result in resource losses, which leads to emotional exhaustion. As for Machiavellian employees, they show great environmental adaptability (Christie and Geis, 1970) so they are likely to perceive less resource losses in face of role conflict. Machiavellian employees can avoid emotional involvement under high degrees of emotional stress and remain calm while resolving sensitive issues or handling awkward scenarios (Singhapakdi, 1993; Wilson et al., 1996), which can protect and maintain emotional resources. In addition, they are good at manipulating others, taking advantage of surrounding resources and skillfully applying interpersonal strategies in interpersonal interactions, so that they can act efficiently even though they are in competitive situations requiring urgent decision making (Christie and Geis, 1970). Machiavellian employees maintain and gain resources through interpersonal manipulation and strategy application. Employees with high Machiavellianism are not easily confused by conflicting situations, and they act rationally by adjusting their emotions and responding actively (Linton and Wiener, 2001; Sendjaya et al., 2016). As such, they are less likely to experience emotional exhaustion. On the contrary, employees with low Machiavellianism may be more sensitive to conflict situations and are more likely to experience emotional exhaustion, because they are vulnerable to resource losses when faced with role conflict. Therefore, we propose:

*Hypothesis 2*: *Machiavellianism moderates the relationship between role conflict and emotional exhaustion, such that this relationship is weaker for employees with high Machiavellianism than for those with low Machiavellianism.*

Although we have argued that the relationship between role conflict and CWB is mediated by emotional exhaustion, we expect that Machiavellianism buffers this indirect relationship. Individuals with high Machiavellianism demonstrate better situational adaptability (Wilson et al., 1996), so they are less likely to feel exhausted or restricted when faced with role conflict and subsequently engage in less CWB. In addition, according to COR theory, employees engage in CWB to obtain resources to make up for resource losses caused by role conflict, whereas employees with high Machiavellianism find it easier to handle conflicting expectations, indicating that they are more capable of protecting their resources. Since employees with high Machiavellianism are better at coping with role conflict, emotional exhaustion and subsequent CWB is less likely to occur. Therefore, we propose:

Hypothesis 3: Machiavellianism moderates the indirect effect of role conflict on CWB via emotional exhaustion, such that the indirect effect is weaker for employees with high Machiavellianism than for those with low Machiavellianism.

## Materials and Methods

### Sample and Procedure

Survey questionnaires were distributed in three manufacturing enterprises, including one state-owned enterprise, one domestic private enterprise, and one international enterprise in Wuhan, China. All the participants were full-time employees who had held their positions for more than 6 months. We contacted the human resources managers and arranged a formal training before their monthly meeting to briefly introduce our academic purpose and highlight the anonymity in our survey. All data were collected through self-reporting. The data were collected in three phases, each at a specific time with 4-week durations between the data collection sessions. The first phase of data collection was conducted right after formal training (i.e., Time 1). The second and third phases occurred 4 weeks (i.e., Time 2) and 8 weeks (i.e., Time 3) after training, respectively. Four-week between-session intervals were chosen as that duration was sufficient for participants to forget the logical relationships between the tested variables, and to try to reduce the effect of common method variance (Podsakoff et al., 2003). At Time 1, we coded all the participants and recorded their cell phone numbers and e-mails. After the entire survey was finished, three participants were selected randomly to receive a gift. We e-mailed the survey to the participants who could not attend the remaining sessions on time and requested that they return their responses electronically.

Three hundred two participants from three manufacturing enterprises took part in the first data collection session (i.e., at Time 1). Eight weeks later (Time 3), 255 valid responses were obtained from the participants. The valid response rate was 84.4%. Of the valid participants, 43.5% were male and 56.5% were female. Regarding the job tenure distribution of the participants, 9.4% had been employed for less than 1 year, 37.6% for 1–3 years, 23.1% for 3–5 years, 17.6% for 5–10 years, and 12.2% for over 10 years.

### Measures

All scales used in this research are well established in the literature. To ensure scale equivalence, we performed back translation (Brislin, 1970). First, we asked two doctoral students who majored in business management to translate the English version of the survey into Chinese. Second, they exchanged the Chinese version and translated it back into English. Third, they discussed and modified the Chinese version based on the back-translated version. Finally, we invited two professors to verify the surveys using their professional experience to ensure that the final Chinese version was clear to understand.

#### Role Conflict (Collected at Time 1)

Role conflict was measured using five items from the Role Questionnaire, which was originally developed by Rizzo et al. (1970). The original scale consisted of two dimensions, role conflict and role ambiguity. We focused on role conflict. The responses were scored on a 5-point Likert scale ranging from 1 (strongly disagree) to 5 (strongly agree). Some of the items used were, “I have to buck a rule or policy to carry out an assignment,” “I receive an assignment without adequate resources and materials to execute it,” and “I work on unnecessary things.” The Cronbach’s alpha for this scale was 0.81.

#### Emotional Exhaustion (Collected at Time 2)

Emotional exhaustion was assessed using nine items from the Maslach Burnout Inventory (Maslach and Jackson, 1981). The responses were scored on a 5-point Likert scale ranging from 1 (strongly disagree) to 5 (strongly agree). Some of the items used were, “I feel emotionally drained from work” and “I feel frustrated by my job.” The Cronbach’s alpha for the emotional exhaustion scale was 0.96.

#### Counterproductive Work Behaviors (Collected at Time 3)

We measured CWB using the workplace deviance scale from Bennett and Robinson (2000), which was divided into two dimensions: organization-focused CWB (13 items) and individual-focused CWB (six items). We noted that one item, “Made an ethnic, religious, or racial remark at work,” was inappropriate in the Chinese context, so we removed it from the formal survey. In contrast to positive behaviors, CWB have a strong sensitivity and is highly concealable, so it is difficult for employees’ colleagues and supervisors to perceive or observe it. Bennett and Robinson (2000) and Jones (2009) suggested that it is more accurate and effective to measure CWB by self-report method. The responses were scored on a 5-point Likert scale ranging from 1 (almost never) to 5 (almost always). Some of the items used were, “take property from work without permission” and “make fun of someone at work.” The Cronbach’s alphas for organization-focused and individual-focused CWB were 0.92 and 0.94, respectively, and the total Cronbach’s alpha was 0.95.

#### Machiavellianism (Collected at Time 1)

We used the Machiavellian Personality Scale with 16 items to measure Machiavellianism. The scale was developed by Dahling et al. (2009). Their conceptualization of Machiavellianism includes dimensions of observable behaviors, internal beliefs and motivation, such as amoral manipulation (five items), desire for control (three items), desire for status (three items), and distrust of others (five items). The responses were scored on a 5-point Likert scale ranging from 1 (strongly disagree) to 5 (strongly agree). Some of the items used were, “I believe that lying is necessary to maintain a competitive advantage over others” and “I like to give the orders in interpersonal situations.” The Cronbach’s alphas for the subscales were 0.85, 0.71, 0.74, and 0.82, respectively, and the total Cronbach’s alpha was 0.87.

#### Control Variable (Collected at Time 1)

We controlled for the possible effects of gender, and tenure on CWB, as research has suggested that they may significantly affect CWB (Lau et al., 2003; Bai et al., 2016). There were two categories for gender: male (1) and female (2). There were five levels for job tenure: less than 1 year (1), 1–3 years (2), 3–5 years (3), 5–10 years (4), and over 10 years (5).

### Analytical Strategy

We first conducted unstandardized ordinary least squares regression to preliminary examine the relationship proposed in our theoretical model. Moreover, as we proposed indirect effect (i.e., Hypothesis 1) and moderated indirect effect (i.e., Hypothesis 3), we adopted moderated path analysis following the recommendation of Edwards and Lambert (2007). Besides, to test the moderating effect of Machiavellianism (Hypothesis 2), we employed the hierarchical regressions to examine the proposed interactive effects. Then, we followed Aiken et al. (1991) recommendation for plotting the interactions.

## Results

### Measurement Model Testing

We used confirmatory factor analysis to test the discriminant validity. Four main variables were used in this analysis: role conflict, emotional exhaustion, CWB, and Machiavellianism. We formed four parcels as indicators for Machiavellianism and two parcels as indicators for CWB by averaged the items into dimensions (Cortina et al., 2001), using the different dimensions as separate indicators of the corresponding constructs (Zhang and Bartol, 2010). Role conflict and emotional exhaustion were analyzed directly in the items (Netemeyer et al., 1990). Against the baseline model of four factors, we examined three alternative models. **Table 1** showed that the fitting effect of the four-factor model (*χ^2^* = 376.08, *df* = 152, TLI = 0.93, CFI = 0.95, GFI = 0.88, SRMR = 0.07, RMSEA = 0.08) was significantly better than the three-factor model, two-factor model, and one-factor model. The results indicate that the four-factor model was better than any of the alternatives, indicating good discriminant validity between each variable. The composite reliability of each construct reached 0.71 or higher. Consequently, the convergent validity was also confirmed. Overall, the four-factor construct was valid and distinct.

**Table 1:** Comparison of alternative factor structure models (*N* = 255).

Models	Factor structures	*x*^2^	*df*	TLI	CFI	GFI	SRMR	RMSEA
Four-factor	Role conflict; emotional exhaustion; CWB; Machiavellianism	376.08	152	0.93	0.95	0.88	0.07	0.08
Three-factor	Emotional exhaustion and Machiavellianism were combined into one factor	509.48	156	0.90	0.92	0.85	0.12	0.09
Two-factor	Role conflict, emotional exhaustion and Machiavellianism were combined into one factor	814.91	158	0.82	0.85	0.73	0.14	0.13
One-factor	All factors combined into one factor	1278.18	162	0.69	0.74	0.70	0.15	0.17


### Descriptive Analysis

**Table 2** presents the descriptive statistics and zero-order correlations of the variables and includes role conflict, emotional exhaustion, Machiavellianism, and CWB. As expected, CWB were significantly positively correlated with role conflict (*r* = 0.40, *p* < 0.01), emotional exhaustion (*r* = 0.26, *p* < 0.01), and Machiavellianism (*r* = 0.49, *p* < 0.01). Overall, the correlation coefficients confirm our hypotheses. Additionally, gender was negatively related to role conflict (*r* = -0.16, *p* < 0.01) and CWB (*r* = -0.15, *p* < 0.01). Tenure was positively related to CWB (*r* = 0.18, *p* < 0.01).

**Table 2:** Descriptive statistics and zero-order correlations (*N* = 255).

Variables	1	2	3	4	5	6
Mean	1.57	2.84	2.47	2.73	2.38	2.07
SD	0.50	1.18	0.81	0.84	0.59	0.72
1. Gender	-					
2. Tenure	-0.12	-				
3. Role conflict	-0.16**	-0.00	-			
4. Emotional exhaustion	0.02	0.01	0.24**	-		
5. Machiavellianism	-0.28**	0.07	0.34**	0.13*	-	
6. CWB	-0.15**	0.18**	0.40**	0.26**	0.49**	-


*^∗^*p* < 0.05, ^∗∗^*p* < 0.01, all two-tailed tests.*

### Hypotheses Testing

**FIGURE 1:**
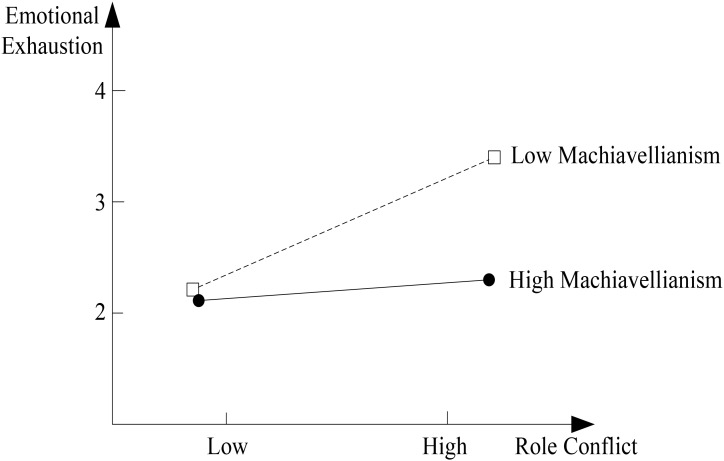
Moderating effect of Machiavellianism on the relationship between role conflict and emotional exhaustion.

We used the Mplus 6.11 to test the hypotheses. The coefficient results are shown in **Table 3**. For Hypotheses 1–3, and we use bootstrap resampling (2,000 times) to test the hypotheses. Hypothesis 1 suggests that emotional exhaustion mediates the relationship between role conflict and CWB. Generally, if the confidence intervals of the results exclude 0, the mediation effect is supported (Preacher et al., 2010). As shown in **Table 3**, role conflict had a significant positive effect on emotional exhaustion (β = 0.24, *p* < 0.01) and emotional exhaustion had a positive effect on CWB (β = 0.13, *p* < 0.05). The mediation effect was 0.03 with a 95% confidence interval of [0.01, 0.07], not including 0. This suggested that role conflict had a positive effect on CWB indirectly through emotional exhaustion, supporting Hypothesis 1. Hypothesis 2 suggests that Machiavellianism moderates the relationship between role conflict and emotional exhaustion. **Table 3** showed that Machiavellianism had a significant moderation effect on the relationship between role conflict and emotional exhaustion (β = -0.35, *p* < 0.01). Additionally, simple slope test suggested that the relationship between role conflict and emotional exhaustion was significantly positive when Machiavellianism was low (β = 0.45, *t* = 4.69, *p* < 0.001), and not significant when Machiavellianism was high (β = 0.03, *t* = 0.29, *n.s.*, see **Figure 1**). The difference between low Machiavellianism and high Machiavellianism was significant (β = -0.42, *t* = -2.69, *p* < 0.01). Thus, Hypothesis 2 was supported. Hypothesis 3 suggests that Machiavellianism moderates the mediation effect of emotional exhaustion between role conflict and CWB. We adopted moderated path analysis, which was introduced by Edwards and Lambert (2007). As shown in **Table 3**, the indirect effects of role conflict on CWB between the low Machiavellianism group and the high Machiavellianism group were different and significant (β = -0.05, 95% CI = [-0.12, -0.01], excluding 0). Thus, Hypothesis 3 was supported.

**Table 3:** Unstandardized ordinary least squares regression coefficients with confidence intervals.

Variables	Emotional exhaustion			CWB		
	Coefficient		95% CI	Coefficient		95% CI
Gender	0.14 (0.11)		[-0.08, 0.32]	0.02 (0.08)		[-0.14, 0.17]
Tenure	0.03 (0.05)		[-0.06, 0.12]	0.09^∗∗^ (0.03)		[0.02, 0.15]
Role conflict	0.24^∗∗^ (0.07)		[0.09, 0.38]	0.20^∗∗∗^ (0.05)		[0.09, 0.30]
Emotional exhaustion				0.13^∗^ (0.05)		[0.03, 0.23]
Machiavellianism	0.15 (0.11)		[-0.10, 0.35]	0.47^∗∗∗^ (0.07)		[0.34, 0.60]
Role conflict × Machiavellianism	-0.35^∗∗^(0.13)		[-0.58, -0.07]			
*R*^2^		0.11^∗∗∗^			0.34^∗∗∗^	
**Machiavellianism**				
Conditional indirect effect of role conflict on CWB	Effect		SE	Boot LL CI (95%)		Boot UL CI (95%)
Low	0.06		0.02	0.02		0.11
High	0.00		0.02	-0.02		0.04
Diff	-0.05		0.03	-0.12		-0.01


*N = 255. Bootstrap = 2000. CI, confidence interval; LL, lower limit; UL, upper limit; Diff, the difference between high and low. ^∗^*p* < 0.05; ^∗∗^*p* < 0.01; ^∗∗∗^*p* < 0.001.*

## Discussion

We developed and integrated a theoretical framework to understand the influence of both job stressors (i.e., role conflict) and personality traits (i.e., Machiavellianism) on CWB. The results confirmed that role conflict has a positive effect on employees’ CWB through the mediating role of emotional exhaustion. Moreover, we found that role conflict and Machiavellianism have a joint effect on emotional exhaustion and CWB. Specifically, in contrast to low Machiavellianism, employees with high Machiavellianism experienced less emotional exhaustion and engaged in less CWB when they were facing role conflict.

### Theoretical and Empirical Contributions

Our research contributes to literature on CWB and Machiavellianism in three ways. First, based on COR theory, we have examined the positive relationship between role conflict and CWB *via* emotional exhaustion in a non-Western setting (i.e., China). Although differences exist between Eastern and Western cultures (e.g., power distance, Hofstede, 1984; traditionality, Farh et al., 2007), we have found consistent results over the relationship between role conflict and CWB, which has increased our knowledge about the antecedents of CWB. Meanwhile, although research has explored the mediating role of personal emotions and attitudes in the relationship between work stressors and CWB, little research has specifically examined how role conflict affects CWB from the emotional exhaustion perspective. For example, Fox et al. (2001) showed that negative emotions mediate the stressor-strain relationship based on a job stress–emotion model. Dalal (2005) stated implicitly that emotional exhaustion may play a mediating role in predicting CWB under job stressors. Thus, consistent with past research, we have found that role conflict causes resource losses, leading to emotional exhaustion and resulting in CWB. We thus contribute to CWB literature by providing evidence that facilitates the understanding of the relationship between job stressors (i.e., role conflict) and CWB through emotional exhaustion.

Second, we have explored the joint effect of job stressors and personality traits on CWB. This contributes to CWB literature by enhancing our understanding of the antecedents of CWB. Although scholars have called for investigations into the joint effect of environmental and individual factors on CWB (Sackett and DeVore, 2001), up until now, only few studies have put effort into it (Colbert et al., 2004; Meurs et al., 2013; Sprung and Jex, 2012; Zhou et al., 2014). For example, Ceschi et al. (2016) found that grit and honesty–humility moderate the relationship between job demands and CWB. The hypothesis and research results in this paper is consistent with Ceschi et al. (2016)’s findings that personality traits play a vital role in coping with job demands. In the present study, we found that Machiavellianism buffers the positive effect of role conflict on CWB *via* emotional exhaustion. On the one hand, according to COR theory, Machiavellian employees experience fewer losses of emotional resources when faced with role conflict due to less personal involvement. On the other hand, they may be more able to cope with resource losses caused by role conflict because of their better environmental adaptability and the skillful use of interpersonal strategies in interpersonal interactions (Christie and Geis, 1970; Nelson and Gilbertson, 1991; Penney et al., 2011). Therefore, Machiavellianism can play a buffering role in the role conflict–exhaustion–CWB link. Thus, our research enhances the current knowledge about the antecedents of CWB.

Third, our research has provided more comprehensive perspective to understanding the effects of Machiavellianism. Studies on Machiavellianism have mainly focused on its negative effects at the workplace, such as lower job satisfaction (Hunt and Chonko, 1984), less organizational citizenship behavior (Liu, 2008; Wolfson, 1981), and more CWB (Tang et al., 2008; Winter et al., 2004). However, despite those negative effects, our research has proposed and found that Machiavellianism has a buffering effect on the role conflict–emotional exhaustion link. Specifically, we have found that employees with high Machiavellianism experience less emotional exhaustion and engage in less CWB while coping with role conflict. Thus, our research provides new insights for understanding Machiavellianism at the workplace.

### Managerial Implications

Our research has several practical implications for managers. CWB is generally considered to be costly at the workplace, so managers in organizations should learn how to eliminate or decrease this kind of behavior. Our research results have indicated that role conflict causes employees to experience emotional exhaustion and provokes their CWB. This means that managers should pay more attention to the requirements for and expectations of their employees. Specifically, they should avoid giving them conflicting requirements. Managers should also be sensitive to employees’ emotional states. If they find that an employee is suffering from emotional exhaustion, an additional management action, such as timely communication and work lightening, may help prevent CWB. Besides, since COR plays a vital role in employees’ coping with role conflict, managers could sustain employees’ resources through resource-based interventions to help employee better cope with role conflict.

Managers should also pay more attention to individuals displaying the Machiavellian traits. Managers tend to treat these employees with mixed attitudes. It is true that they can easily undermine management ethics and do things that are unethical or counterproductive. However, they can also be outstanding staff members and accomplish their tasks, while aligning well with organizational goals (Dahling et al., 2009; Karkoulian et al., 2009). The results of this research have shown that employees with high Machiavellianism are better at coping with work stressors and engage less in CWB under stressful conditions. Therefore, managers should avoid judging Machiavellian employees as “bad apples” and try to develop a more complete understanding of them.

### Limitations and Future Research Directions

Despite its contributions, our research does have its share of limitations. First, all the data in our research were collected from self-reports, which may have led to some common method bias. Generally, CWB is not easily recognized and individuals are reluctant to share such behavior with others. This was the reason for our following the suggestions of Bennett and Robinson (2000) and Jones (2009) while measuring CWB through self-reports. However, although we had set time intervals to collect data to reduce common method bias, it may still have limited our research; we could not avoid it entirely. Second, we had conducted the survey in three manufacturing enterprises, which may have limited the generalizability of our results to other industries. Work conditions and CWB in different industries may not be the same (Grijalva and Newman, 2015). For example, employees in service enterprises are usually faced with more complex role expectations and engage in more emotional labor. Thus, future research should test our model in other industries, such as the service industry, to further examine the relationship between work conditions and CWB. Furthermore, although our research examined the relationship between role conflict, emotional exhaustion, and CWB in a non-Western culture (i.e., China), we did not provide much information about whether this relationship would be different across varying cultures. For example, since employees with high power distance will be more likely to obey supervisors’ expectations unconditionally (Farh et al., 2007), it may be possible that this relationship will be weaker in low power distance culture. Thus, it is worthwhile for future researchers to conduct a cross-culture comparison study to examine whether there is a culture difference.

## Conclusion

Machiavellian employees cope better with role conflict by engaging less in CWB. By examining the joint effect of role conflict and Machiavellianism on emotional exhaustion and CWB, we have enhanced the understanding of the interaction effect between job stressors and personality traits on CWB. We have also demonstrated the important mediation role of emotional exhaustion on the relationship between role conflict and CWB. Given our findings, we hope to inspire more research on the joint effect of job stressors and personality traits on CWB, especially that exploring the potential positive effects of Machiavellianism at the workplace.

## Ethics Statement

An ethics approval was not required as per our institution’s guidelines and national regulations. Written informed consent was obtained from all participants in our study.

## Author Contributions

JZ and JM designed and adopted the study, and wrote the paper. SX and WL wrote the paper.

## Conflict of Interest Statement

The authors declare that the research was conducted in the absence of any commercial or financial relationships that could be construed as a potential conflict of interest.
